# Supplementary material for: Social state gates vision using three circuit mechanisms in Drosophila
Source: bioRxiv. 2024 Mar 17:2024.03.15.585289. Preprint. [Version 1] doi: 10.1101/2024.03.15.585289 (PMC10979952; doi:10.1101/2024.03.15.585289)
Supplement: Supplement 1 [file NIHPP2024.03.15.585289v1-supplement-1.pdf]

bioRxiv preprint doi: https://doi.org/10.1101/2024.03.15.585289; this version posted March 17, 2024. The copyright holder for this preprint (which was not certified by peer review) is the author/funder, who has granted bioRxiv a license to display the preprint in perpetuity. It is made available under aCC-BY 4.0 International license.

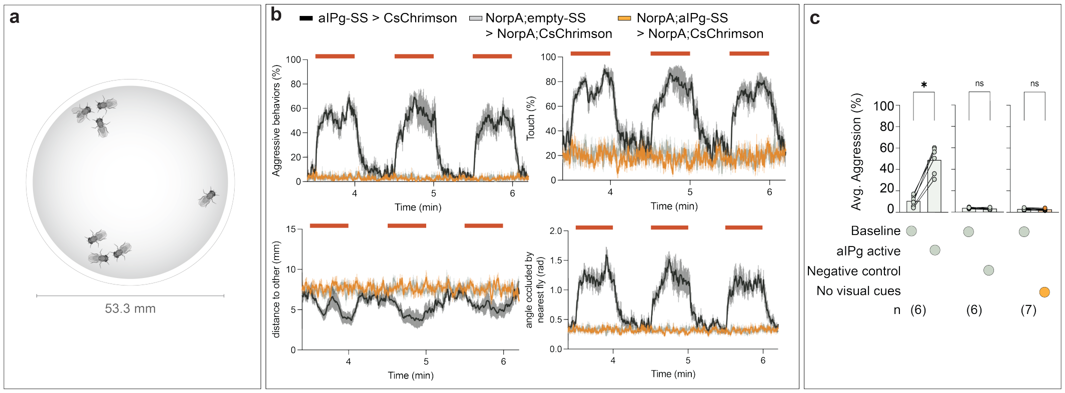

Fig. S1. Pathways carrying visual information are important for female aggressive behaviors and related behavioral features. (a) Diagram of the arena used for female behavioral experiments. We performed behavioral experiments in a standardized 53.3 mm arena in which freely moving fly behavior was quantified using a 170 frames per second camera and computer vision-based classification methods (62, 64). (b) Percentage of flies engaging in aggressive behaviors, touching, and changes in parameters related to distance to another fly and the maximum angle of the field of view occluded by the closest fly (angle occluded by nearest fly) are plotted over the course of a 3.3 min trial during which a 3x 30 s 3 mW/cm^2 continuous red-light stimulus (red bars) were delivered. Prior data are not shown as no significant changes were found in the no visual cues group during this low stimulus period (1 mW/cm^2) as well. The mean is represented as a solid line and shaded bars represent standard error between experiments. The timeseries shows the percentage of flies performing aggression displayed as the mean of 2.83 s (60-frame) bins. (c) Average time spent performing aggressive behaviors before and during stimulus periods. All data points are shown to indicating the range and top edge of bar represents the mean. Each dot represents one experiment containing approximately seven flies. Data supporting the plots shown in panels b – c were as follows: aIPg-SS > CsChrimson, n = 6 experiments; norpA, EmptySS > norpA, CsChrimson, n = 6 experiments; norpA, aIPg-SS > norpA, CsChrimson, n = 7 experiments. Data are representative of two independent replicates, which included separate parental crosses and were collected on different days. A non-parametric Wilcoxon Matched-pairs Signed Rank test was used for statistical analysis. Asterisk indicates significance from 0: *p<0.05.

bioRxiv preprint doi: https://doi.org/10.1101/2024.03.15.585289; this version posted March 17, 2024. The copyright holder for this preprint (which was not certified by peer review) is the author/funder, who has granted bioRxiv a license to display the preprint in perpetuity. It is made available under aCC-BY 4.0 International license.

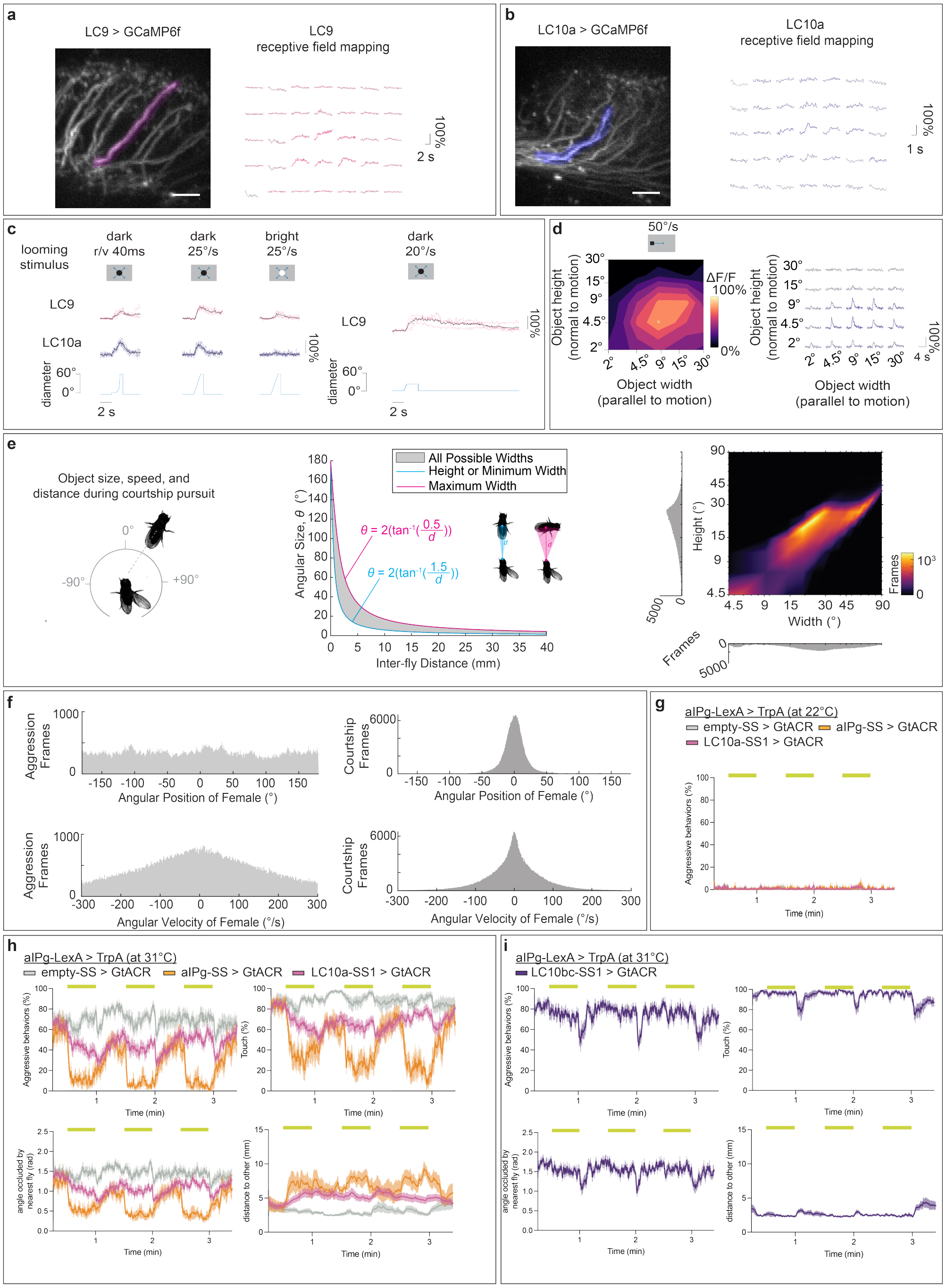

Fig. S2. LC visual feature detection and involvement in female aggression. Panels a – d show the receptive fields for LC9 and LC10a. Panels e – f show the visual experience during male courtship and female aggression. Panels g – i demonstrate the effects of inhibiting LC10a or LC10bc during aIPg activation. (a – b) Single receptive-field mapping for individual LC axons in representative flies. LC9 and LC10a axonal regions of interest are colored in magenta and blue, respectively and overlaid on averaged calcium image (left; Scale bar: 10 μm). Individual calcium responses, arranged as in **Figure 2a**, are shown on right. (c) Single-cell (color) and population average (black) calcium traces for neurons responding to looming stimuli centered on the receptive field, same as performed in (23). LC9: n = 4 flies, n = 4 neurons. LC10a: n = 5 flies, n = 7 neurons (25°/s constant edge speed looming was only recorded for 2 neurons from 1 fly). (D) Size tuning, as measured and plotted in **Figure 2a**, for objects of varying sizes moving at a slower speed of 50°/s. (e – f) (e) Left: histograms show conspecific angular position in the visual field as experienced by the male during courtship pursuit. Visual parameters were calculated from single choice courtship assays (n = 13). During male courtship pursuit, the mean conspecific size as subtended on the retina was 15.96 +/- 4.4° (mean +/- standard deviation) in height and 28.699 +/- 12.7° in width. Right: all possible angular heights and widths for a female with a minor axis of 1 mm and major axis of 3 mm are plotted on the left, and the measured angular sizes and heights across frames in the courtship assays are shown in heat map and histogram representations on far right. (f) Histograms of angular position and velocity during during female aggression (left) and naturalistic male courtship pursuit (right). (g – i) Percentage of flies engaging in behaviors (aggression, touch) or behavioral features (distance to other, angle occluded by nearest fly) over the course of a trial during which 3x 30 s continuous green light (yellow bars) were delivered. To control for additional cell types in the LexA line used for aIPg (75), we simultaneously inhibited aIPg during thermogenetic activation through using an aIPg-specific split-GAL4 line and the green light gated anion channel, GtACR. The dramatic reduction in female aggressive behavior during optogenetic inhibition confirmed that aIPg was primarily responsible for the aggression observed when stimulating the LexA line. Data supporting the plots shown in panels g – i were as follows: g: aIPg-LexA > TrpA emptySS > GtACR, n = 10 experiments; aIPg-LexA > TrpA aIPg-SS > GtACR, n = 8 experiments; aIPg-LexA > TrpA LC10a-SS > GtACR, n = 13 experiments. h: aIPg-LexA > TrpA emptySS > GtACR, n = 15 experiments; aIPg-LexA > TrpA aIPg-SS > GtACR, n = 8 experiments; aIPg-LexA > TrpA LC10a-SS > GtACR, n = 18 experiments. i: aIPg-LexA > TrpA LC10bc-SS > GtACR, n = 11 experiments. Experiments were performed at the permissive temperature (31°C, h – i) for aIPg > TrpA stimulation, and non-permissive temperature controls (22°C) are shown in g. The mean is represented as a solid line and shaded bars represent standard error between experiments. The timeseries shows the percentage of flies performing aggression displayed as the mean of 2.83 s (60-frame) bins.

bioRxiv preprint doi: https://doi.org/10.1101/2024.03.15.585289; this version posted March 17, 2024. The copyright holder for this preprint (which was not certified by peer review) is the author/funder, who has granted bioRxiv a license to display the preprint in perpetuity. It is made available under aCC-BY 4.0 International license.

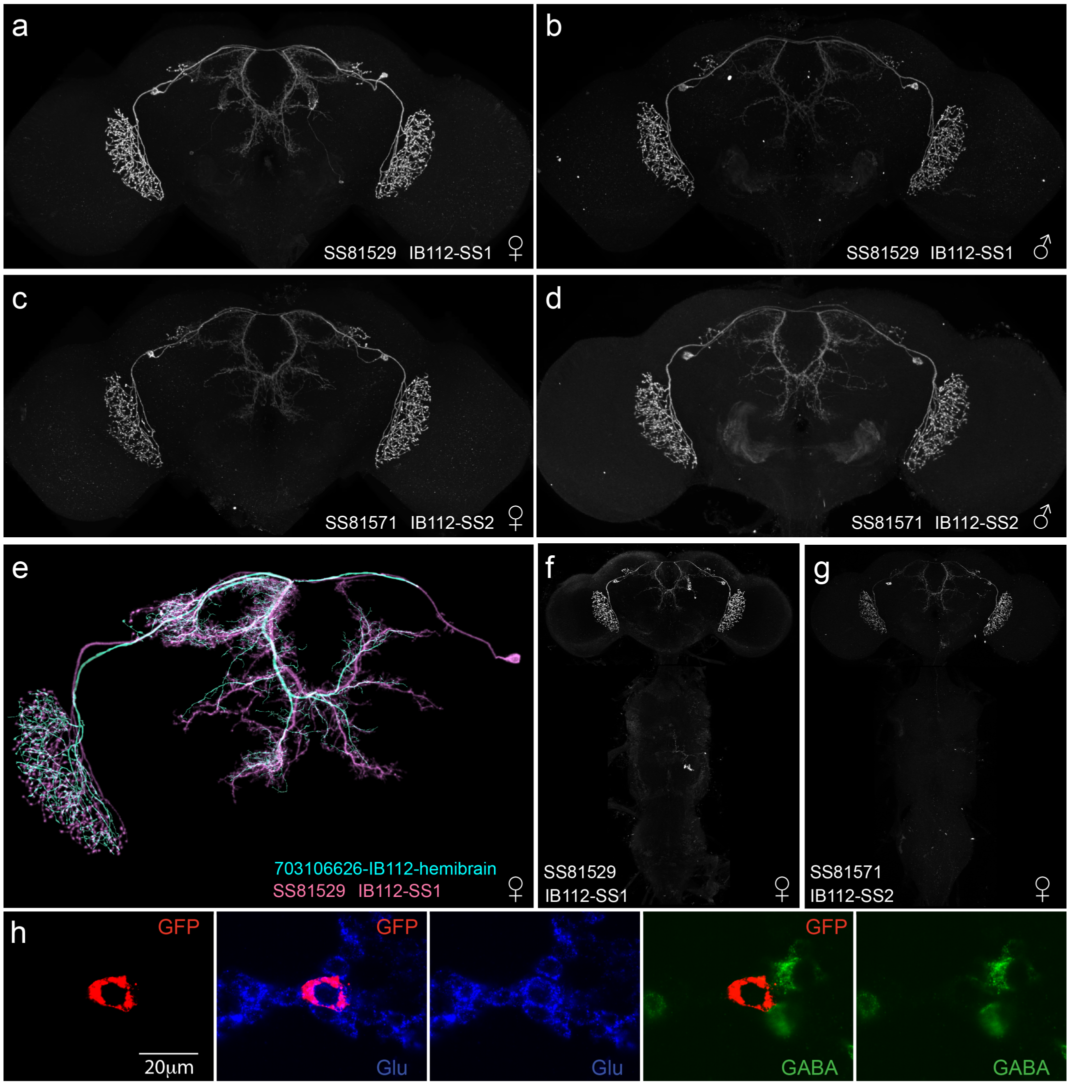

Fig. S3. Anatomy of GAL4 driver lines for IB112. (a, b) Expression patterns in a female and male brain, respectively, of GAL4 line SS81529 (IB112-SS1). (c, d) Expression patterns in a female and male brain, respectively, of GAL4 line SS81571 (IB112-SS2). (e) IB112 body ID 703106626 skeleton from hemibrain v1.2.1 shown together with a neuron from SS81529 obtained by stochastic labeling (70) and then segmented using VVD (see Key Resources table). (f, g) Images of the expression patterns in the brain and VNC of GAL4 driver lines SS81529 and SS81571, as indicated. (h) Images of fluorescent in situ hybridization assays to determine the neurotransmitter used by IB112. Probes used in each panel are indicated. GFP shows the IB112 cell body and Glu and GABA represent probes for vGlut and GAD, respectively (see Methods for details). Scale bar is shown in the left panel.

bioRxiv preprint doi: https://doi.org/10.1101/2024.03.15.585289; this version posted March 17, 2024. The copyright holder for this preprint (which was not certified by peer review) is the author/funder, who has granted bioRxiv a license to display the preprint in perpetuity. It is made available under aCC-BY 4.0 International license.

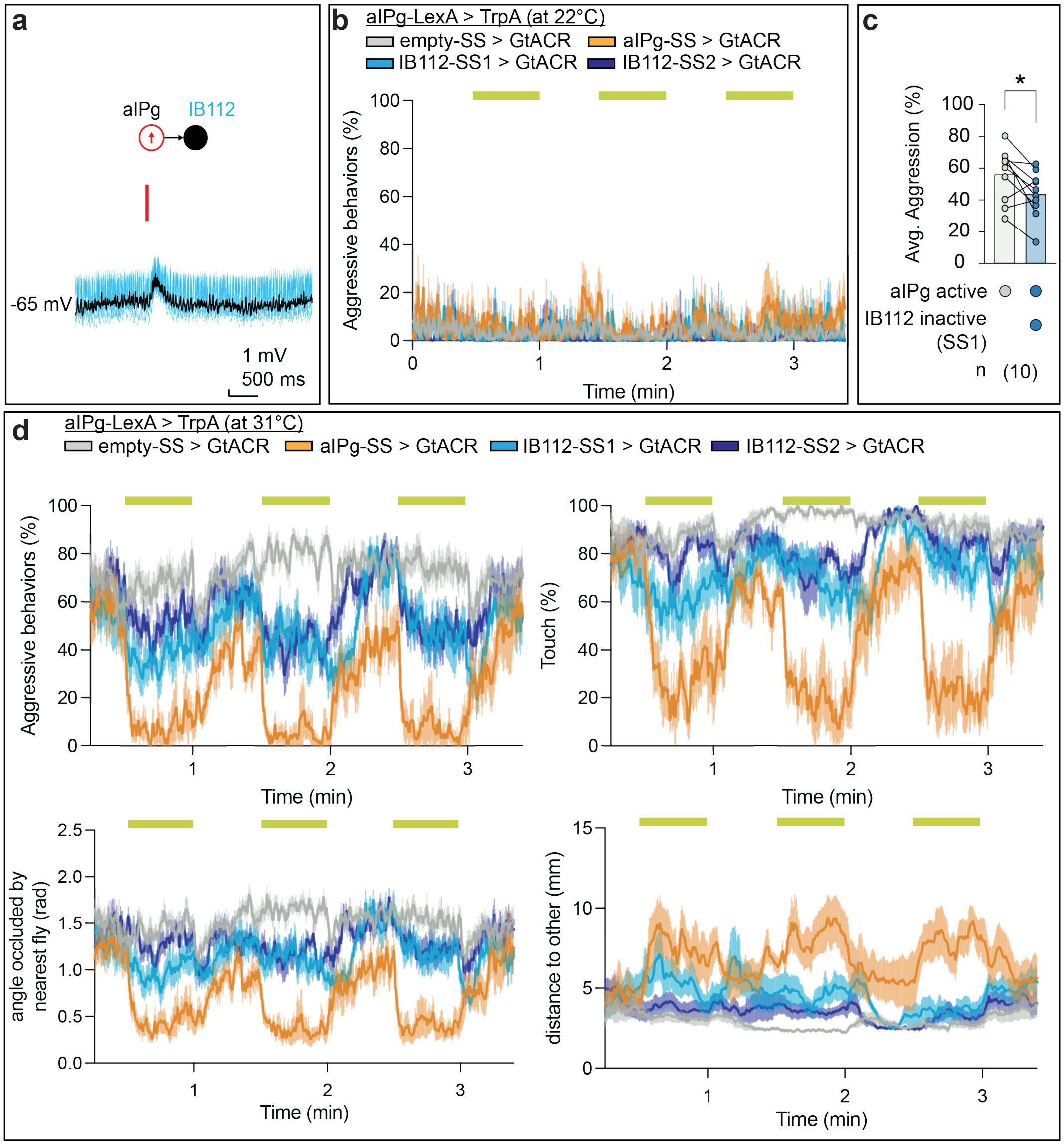

Fig. S4. IB112 shapes aIPg-mediated female aggressive behaviors. (a) Excitatory responses recorded by patch clamp electrophysiology in female brain explants from IB112 (n = 6 cells) before, during, and following a 15 ms activation of aIPg. Individual trials in blue (n = 8 trials from one cell), mean shown in black. (b, d) Percentage of flies engaging in aggression, touch, or changes in related parameters, including the maximum angle of the field of view occluded by the closest fly (angle occluded by nearest fly) or distance to another fly. Percentages are plotted over the course of a trial during which 3x 30 s 9 mW/cm^2 continuous light stimuli (yellow bars) were delivered. The mean is represented as a solid line and shaded bars represent standard error between experiments. The timeseries shows the percentage of flies performing aggression displayed as the mean of 2.83 s (60-frame) bins. (c) Average time spent performing aggressive behaviors before and during stimulus periods. Averages were calculated over all flies in an experiment, and each dot represents one experiment containing approximately seven flies. All data points are shown to indicating the range and top edge of bar represents the mean. Data supporting the plots shown in panels b – d were as follows: b: aIPg-LexA > TrpA emptySS > GtACR, n = 11 experiments; aIPg-TrpA > CsChrimson aIPg-SS > GtACR, n = 5 experiments; aIPg-TrpA > CsChrimson IB112-SS1 > GtACR, n = 4 experiments; aIPg-TrpA > CsChrimson IB112-SS2 > GtACR, n = 4 experiments. c – d: aIPg-LexA > TrpA emptySS > GtACR, n = 13 experiments; aIPg-TrpA > CsChrimson aIPg-SS > GtACR, n = 6 experiments; aIPg-TrpA > CsChrimson IB112-SS1 > GtACR, n = 10 experiments; aIPg-TrpA > CsChrimson IB112-SS2 > GtACR, n = 11 experiments. Experiments were performed at the permissive temperature (31°C, c – d for aIPg > TrpA stimulation, and non-permissive temperature controls (22°C) are shown in b. Data were pooled from four independent replicates, which included separate parental crosses and were collected on different days. A non-parametric Wilcoxon Matched-pairs Signed Rank test was used for statistical analysis. Asterisk indicates significance from 0: *p<0.05.

bioRxiv preprint doi: https://doi.org/10.1101/2024.03.15.585289; this version posted March 17, 2024. The copyright holder for this preprint (which was not certified by peer review) is the author/funder, who has granted bioRxiv a license to display the preprint in perpetuity. It is made available under aCC-BY 4.0 International license.

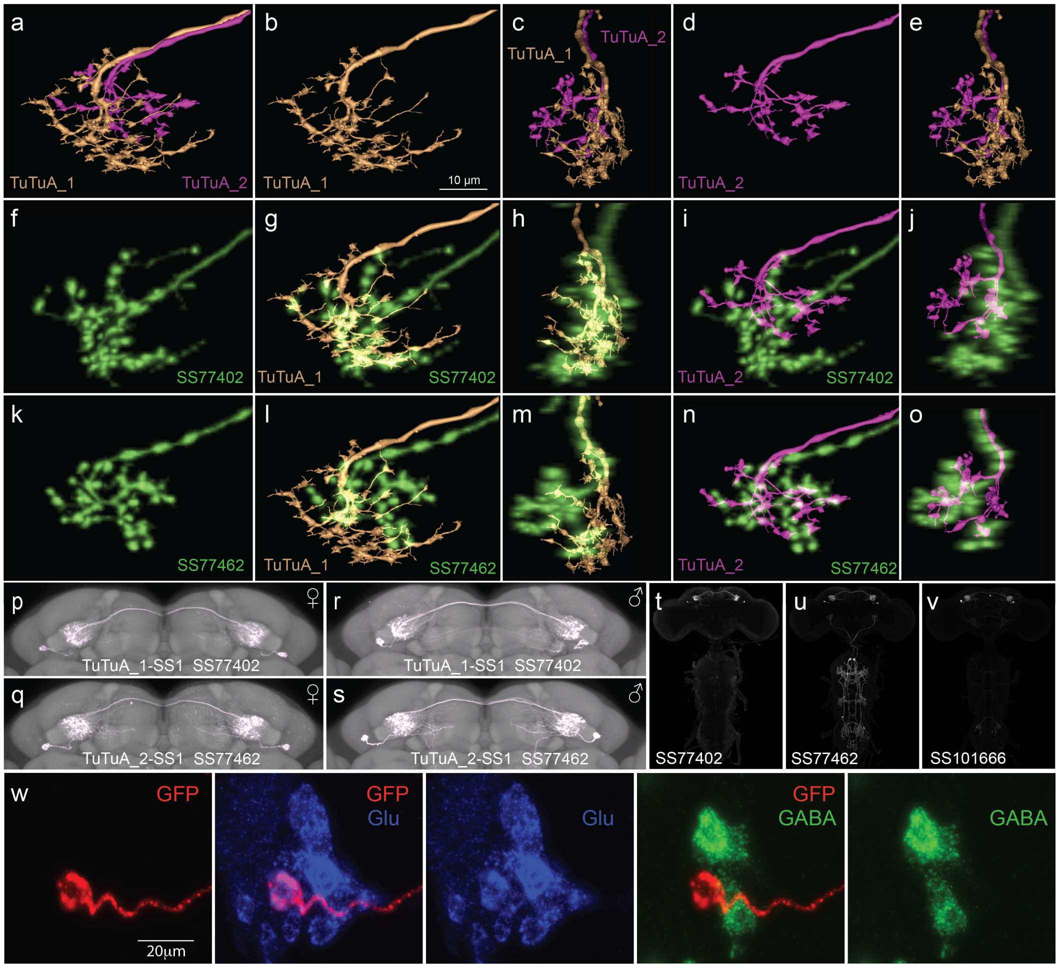

Fig. S5. Anatomy of TuTuA subtypes. (a – e) Neuronal skeletons of the termini of the contralateral axons of TuTuA subtypes from the hemibrain v1.2.1 connectome. (a) TuTuA_1 and TuTuA_2 are shown together (body IDs 676836779 and 5813013691, respectively). (b) TuTuA_1 (body ID 676836779) shown alone. (c) Same as panel a, but rotated 90 degrees along the medial-lateral axis. (d) TuTuA_2 (body ID 5813013691) shown alone. (e) Same as panel c, repeated to facilitate comparison. These anatomical differences were used to determine the correspondence between GAL4 driver lines and TuTuA subtypes. (f) Terminus of a contralateral axon of a neuron from GAL4 driver line SS77402 obtained by stochastic labeling(70) and then segmented using VVD (see Key Resources table). (g) The comparison between the GAL4 driver line in f to TuTuA_1 skeleton shown in b. (h) Same as panel g, but rotated 90 degrees along the medial-lateral axis. (i) The comparison between the GAL4 driver line in f to TuTuA_2 skeleton shown in d. (j) Same as panel i but rotated 90 degrees along the medial-lateral axis. (k) Terminus of a contralateral axon of a neuron from GAL4 driver line SS77462 obtained by stochastic labeling(70) and then segmented using VVD. (l) The comparison between the GAL4 driver line in k to TuTuA_1 skeleton shown in b. (m) Same as panel g, but rotated 90 degrees along the medial-lateral axis. (n) The comparison between the GAL4 driver line in k to TuTuA_2 skeleton shown in d. (o) Same as panel i, but rotated 90 degrees along the medial-lateral axis. (p, r) Images of GAL4 driver line SS77402 in females and males, respectively, shown with the standard neuropil reference, JFRC2018U (76) (q, s) Images of GAL4 driver line SS77462 in females and males, respectively. Note the presence of a single TuTuA cell body in each brain hemisphere. (t – v) Images of the expression patterns in the brain and VNC of GAL4 driver lines SS77402, SS77462 and SS10166, as indicated. (w) Images of fluorescent in situ hybridization assays to determine the neurotransmitter used by TuTuA. Probes used in each panel are indicated. GFP shows the TuTuA cell and Glu and GABA represent probes for vGlut and GAD, respectively (see Methods for details). Scale bar is shown in the left panel.

bioRxiv preprint doi: https://doi.org/10.1101/2024.03.15.585289; this version posted March 17, 2024. The copyright holder for this preprint (which was not certified by peer review) is the author/funder, who has granted bioRxiv a license to display the preprint in perpetuity. It is made available under aCC-BY 4.0 International license.

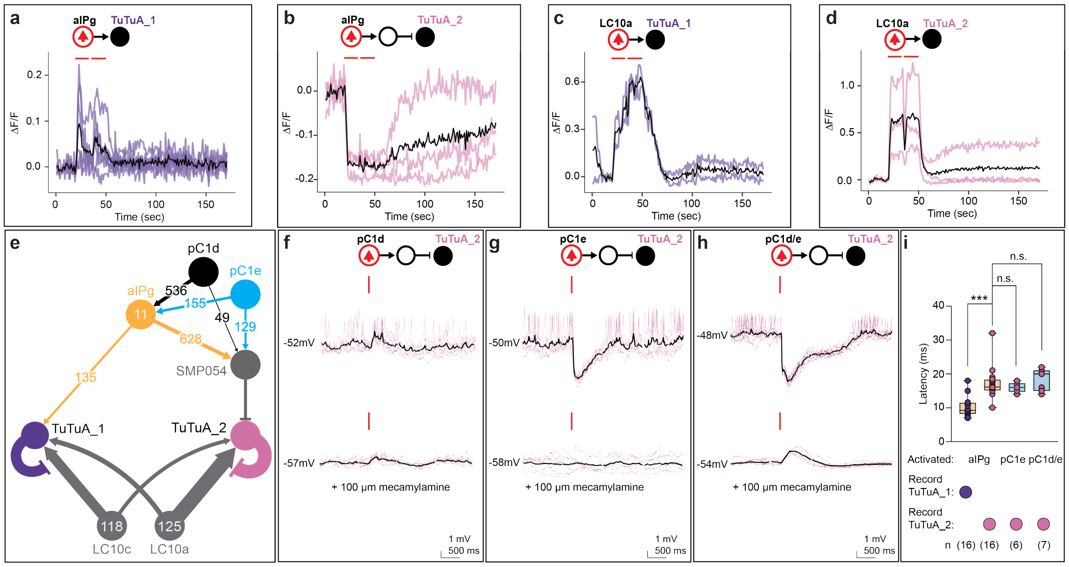

Fig. S6. Responses of TuTuA subtypes to the activation of female aggression or LC10a cell types. (a – d) Changes in florescence intensity as measured by GCaMP6f in the cell body of TuTuA_1 (a, c) or TuTuA_2 (b, d) before, during, and following two 14 s stimuli (2 s interval) at 10 Hz. Individual trials for a – d are shown in purple (TuTuA_1) or pink (TuTuA_2), mean is in black. (e) Connectivity diagram from **Figure 4a** with the connections from pC1d and pC1e. Exact synapse numbers are indicated on the arrows, which are also scaled according to synapse counts. Arrows indicate putative excitatory connections (cholinergic) and bar endings indicate putative inhibitory connections (SMP054, GABAergic; TuTuA_1 and TuTuA_2, glutamatergic). (f – h) Electrophysiology recordings with the cell types activated with CsChrimson are circled in red, and those recorded are in black. Individual trials are in pink (n = 8 trials from 1 cell), mean is in black. (f) Small excitation or negligible response in TuTuA_2 (n = 5 cells) to 15 ms pC1d activation, which was abolished by mecamylamine. (g) Large inhibitory response in TuTuA_2 to 15 ms pC1e activation, which was abolished by mecamylamine (n = 6 cells). (h) Large inhibitory response in TuTuA_2 to 15 ms pC1d/e activation, which was abolished by mecamylamine (n = 7 cells). (i) Latency after stimulus onset (ms). Box-and-whisker plots show median and IQR; whiskers show range. A Kruskal-Wallis and Dunn's post hoc test was used for statistical analysis. Asterisk indicates significance from 0: ***p<0.001.

bioRxiv preprint doi: https://doi.org/10.1101/2024.03.15.585289; this version posted March 17, 2024. The copyright holder for this preprint (which was not certified by peer review) is the author/funder, who has granted bioRxiv a license to display the preprint in perpetuity. It is made available under aCC-BY 4.0 International license.

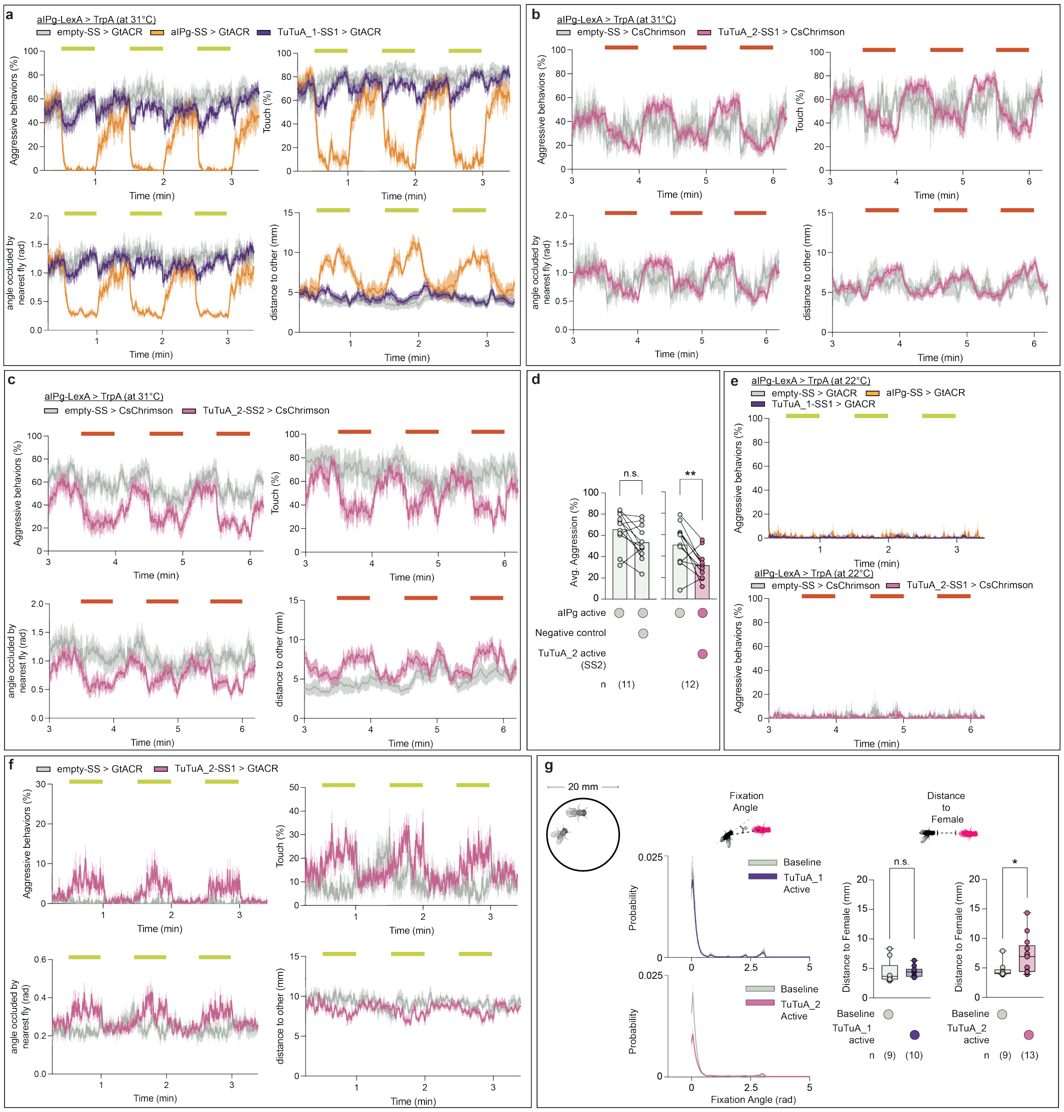

Fig. S7. The TuTuA switch shapes female aggression and male courtship behaviors. (a – c, e – f) Percentage of flies engaging in behaviors (aggression, touch) or behavioral features (distance to other, angle occluded by nearest fly) over the course of a trial during which 3x 30 s continuous light stimuli (yellow or red bars) were delivered. Experiments were performed at the permissive temperature (31°C, a – c) for aIPg > TrpA stimulation, and non-permissive temperature controls (22°C) are shown in e. (d) Percentage of flies engaging in aggression over the course of a 3.3 min trial during which 3x 30 s continuous 9 mW/cm^2 green light (yellow bars) were delivered. Data were pooled from three independent replicates, which included separate parental crosses and were collected on different days. Data supporting the plots shown in panels a – f were as follows: a: aIPg-LexA > TrpA emptySS > GtACR, n = 12 experiments; aIPg-LexA > TrpA aIPg-SS > GtACR, n = 7 experiments; aIPg-LexA > TrpA TuTuA_1-SS > GtACR, n = 20 experiments. b: aIPg-LexA > TrpA emptySS > CsChrimson, n = 6 experiments; aIPg-LexA > TrpA TuTuA_2-SS1 > CsChrimson, n = 18 experiments. c, d: aIPg-LexA > TrpA emptySS > CsChrimson, n = 11 experiments; aIPg-LexA > TrpA TuTuA_2-SS2 > CsChrimson, n = 12 experiments. e (top panel): aIPg-LexA > TrpA emptySS > GtACR, n = 11 experiments; aIPg-LexA > TrpA aIPg-SS > GtACR, n = 4 experiments; aIPg-LexA > TrpA TuTuA_1-SS, n = 11 experiments. e (bottom panel): aIPg-LexA > TrpA emptySS > CsChrimson, n = 4 experiments; aIPg-LexA > TrpA TuTuA_2-SS1 > CsChrimson, n = 9 experiments. f: emptySS > GtACR, n = 6 experiments; TuTuA_2-SS1 > GtACR, n = 11 experiments. The mean for a – c and d - f is represented as a solid line and shaded bars represent standard error between experiments. The timeseries shows the percentage of flies performing aggression displayed as the mean of 2.83 s (60-frame) bins. For figures b – c and the bottom panel of e, data from the low stimulus periods (1 mW/cm^2) prior are not shown as no significant changes were found. Averages were calculated over all flies in an experiment, with each dot representing one experiment containing approximately seven flies. All data points are shown to indicating the range and top edge of bar represents the mean. (g) Facing angle and average distance to female flies during a male-female pair courtship assay. Diagram of the arena used for courtship experiments shown in inset image on the left. The following genotypes were used: (Left panel) Baseline (TuTuA_1-SS1 > CsChrimson, without retinal), TuTuA_1 active (TuTuA_1-SS1 > CsChrimson, with retinal); (Right panel) Baseline (TuTuA_2-SS1 > CsChrimson, without retinal), TuTuA_2 active (TuTuA_2-SS1 > CsChrimson, with retinal). Box-and-whisker plots show median and IQR; whiskers show range. A non-parametric Wilcoxon Matched-pairs Signed Rank test (d) or Mann-Whitney test (g) was used for statistical analysis and each dot represents one pair in g. Asterisk indicates significance from 0: *p<0.05; **p<0.01.

bioRxiv preprint doi: https://doi.org/10.1101/2024.03.15.585289; this version posted March 17, 2024. The copyright holder for this preprint (which was not certified by peer review) is the author/funder, who has granted bioRxiv a license to display the preprint in perpetuity. It is made available under aCC-BY 4.0 International license.

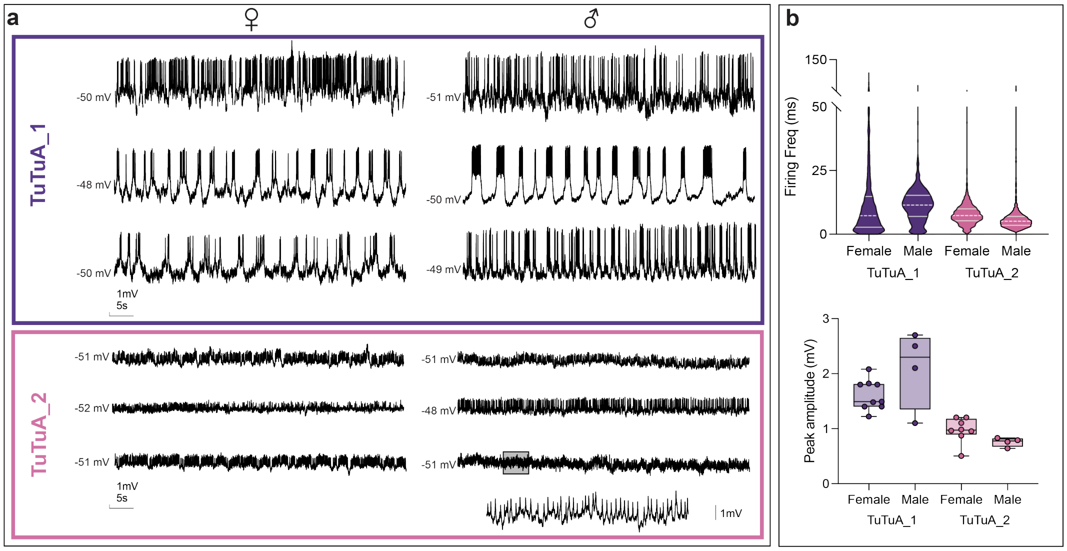

Fig. S8. Recordings from TuTuA_1 and TuTuA_2 in males and females. Each trace is one-minute recording from one cell. TuTuA_1 displayed the larger action potential amplitude compared to TuTuA_2, with similar properties between males and females. Inset recording from a TuTuA_2 neuron is from the highlighted region of the last male recording. (b) Analysis of the firing frequency and peak amplitude of TuTuA_1 and TuTuA_2 recordings in males and females. The instantaneous action potential frequency was calculated for about one minute in each cell (TuTuA_1: Female, n = 1536, Male, n = 1965; TuTuA_2: Female, n = 1198, Male, n = 1185). The action potential amplitude was averaged from 20-30 individual events in each cell (each dot represents 1 cell), and measured as the difference between the threshold and peak (TuTuA_1: Female, n = 9, Male, n = 4; TuTuA_2: Female, n = 8, Male, n = 4). The firing frequency was more variable in the TuTuA_1 recordings than in the TuTuA_2 recordings in both males and females. Additionally, the amplitude from TuTuA_1 was larger compared to TuTuA_2 in both males and females. However, the action potential is dramatically slower in male TuTuA_2 neurons. Box-and-whisker and violin plots show median and IQR; whiskers or ends of the violin show range.

bioRxiv preprint doi: https://doi.org/10.1101/2024.03.15.585289; this version posted March 17, 2024. The copyright holder for this preprint (which was not certified by peer review) is the author/funder, who has granted bioRxiv a license to display the preprint in perpetuity. It is made available under aCC-BY 4.0 International license.

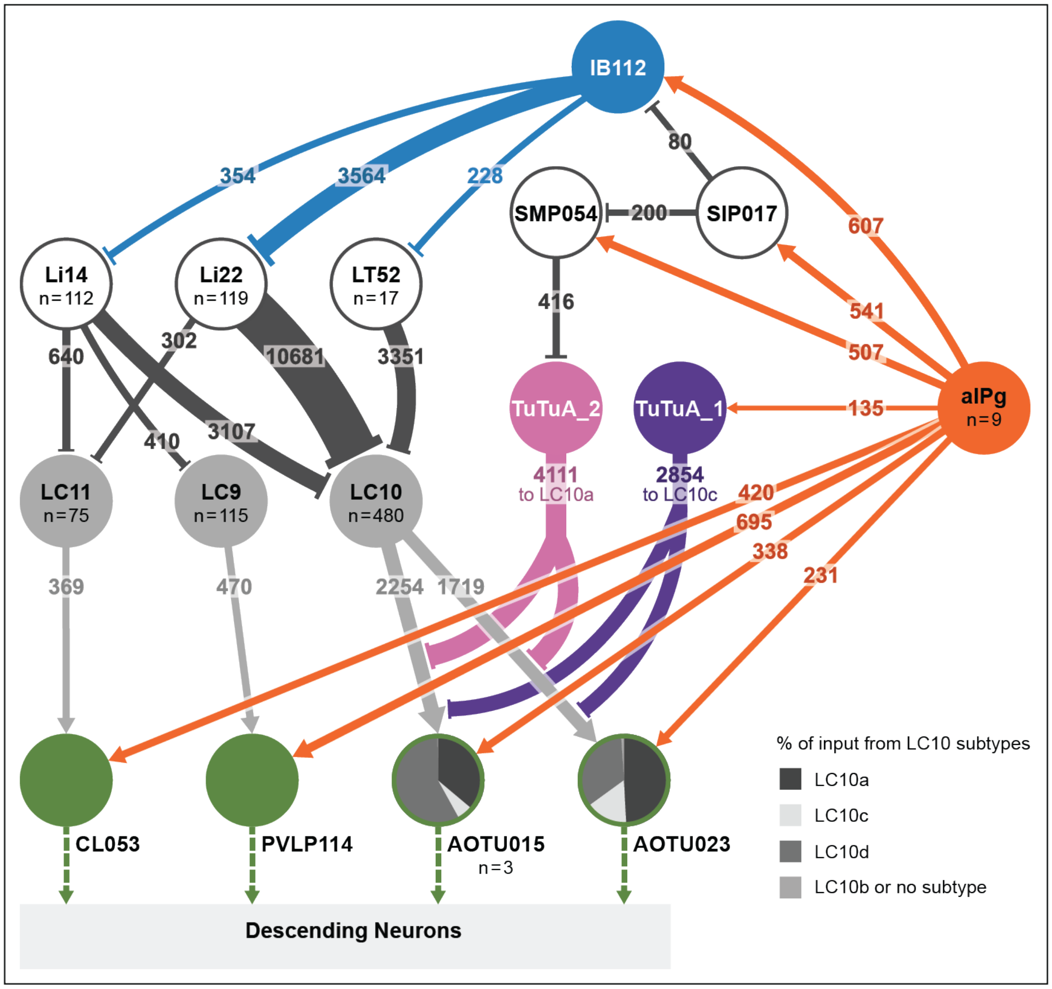

Fig. S9. Circuit diagram of aIPg modulation of visual processing. A detailed circuit map of the mechanisms detailed in Figure 1a and Figure 6. This diagram shows additional downstream targets of aIPg including those involved in regulating information flow from LC9 and LC11. The diagram also illustrates that CL053, PVLP114, AOTU015 and AOTU023 are each upstream of descending interneurons (DNs) that traverse the neck into the ventral nerve cord where they likely regulate motor action. Each of these neurons connect to largely non-overlapping sets of DNs, implying that these parallel pathways control different motor actions. Numbers within arrows indicate synapses numbers. The top six downstream targets of aIPg are represented in this diagram: (1) PVLP114; (2) IB112; (3) SMP054; (4) SIP017; (5) CL053; and (6) AOTU015. Li22 devotes 66% (10,627/16,168) of its synapses going to any LC cell type to LC10 and provides input to all LC10 subtypes; on average the number of Li22 inputs per cell to each LC10 subtype are as follows: LC10a, 23; LC10b, 12; LC10c, 27; and LC10d, 32. Li14 distributes its output more broadly with only 20% of its output to LC neurons going to LC10 with a more biased distribution between subtypes than Li22; on average the number of Li14 inputs per cell to each LC10 subtype are as follows: LC10a, 14; LC10b, 25; LC10c, 2; and LC10d, 5. LT52 devotes 57% of its synapses in the lobula that go to LC neurons to LC10 subtypes with a strong bias to LC10b and LC10d; on average the number of LT52 inputs per cell to each LC10 subtype are as follows: LC10a, 5; LC10b, 27; LC10c, <1; and LC10d, 12.
